# Unlocking iron: nutritional origins, metabolic pathways, and systemic significance

**DOI:** 10.3389/fnut.2025.1637316

**Published:** 2025-08-13

**Authors:** Zoha Imtiaz Malik, Muhammad Umer Ghafoor, Syed Hassan Bin Usman Shah, Juweria Abid, Umar Farooq, Abdul Momin Rizwan Ahmad

**Affiliations:** ^1^Department of Public Health, Health Services Academy, Islamabad, Pakistan; ^2^Al Sadd Health Centre, Primary Healthcare Corporation, Doha, Qatar; ^3^The Kirby Institute, University of New South Wales, Sydney, NSW, Australia; ^4^Department of Nutrition and Dietetics, National University of Medical Sciences (NUMS), Rawalpindi, Pakistan; ^5^Department of Health Sciences, University of York, York, United Kingdom; ^6^Department of Human Nutrition and Dietetics, NUST School of Health Sciences, National University of Sciences and Technology (NUST), Sector H-12, Islamabad, Pakistan

**Keywords:** systemic regulation of iron, assessment indicators, iron absorption, iron homeostasis, heme and non-heme iron

## Abstract

Iron is a vital micronutrient, involved in numerous cellular functions including DNA synthesis, oxygen transport, energy generation, and immunity enhancement. It has unique redox properties that make it necessary for multiple metabolic reactions. However, the same property warrants its tight regulation as well. Despite its widely acknowledged role in biological sciences, iron deficiency remains one of the leading micronutrient deficiencies across the globe. Understanding iron’s multifaceted roles, mechanisms of systemic regulation, and nutriture assessment, can better guide public health interventions aimed at restoring iron status in at-risk population groups. This review aims at providing a comprehensive analysis of iron’s dietary sources, metabolic role, physiological functioning, absorption enhancers and inhibitors, and systemic regulation, such as hepcidin’s role in iron homeostasis. It further evaluates commonly utilized iron assessment indicators including serum ferritin, transferrin saturation, soluble transferrin receptor, and hemoglobin their uses and limitations, particularly in the context of inflammation. Iron’s metabolic roles are closely tied to its bioavailability and transportation within the body. Nutrient interactions, regulator pathway dysregulation, and dietary pathways, all significantly affect iron status. Available iron assessment indicators are valuable but research is required to interpret findings in inflammatory states. Advancement of integrated iron assessment strategies and metabolic roles can help address iron-related disorders more effectively.

## 1 Introduction

Iron makes up about 5% of the earth’s crust by weight and is one of the metabolically active micronutrients. The redox states of iron make it very beneficial with regards to the evolution of biological processes (1). Iron is an essential component to every living being on earth as it serves to be an integral part of several metabolic functions (2). It is an extremely important nutrient for human survival as it is one of the major constituents of hemoglobin (3). Iron is vital for the occurrence of various biological functions such as respiration, synthesis of DNA, proliferation of cells and production of cellular energy. In addition to being an essential element for the synthesis of hemoglobin, it is also a vital component of approximately 200 enzymes, which are required for optimal cellular functions (4). Moreover, iron also serves as a catalyst for different biochemical reactions. Various studies have recently suggested that iron has a definite role to play in some of the vital functions performed by the central nervous system which includes myelination of the nerves as well as synthesis of neurotransmitters (5).

Iron has long been recognized as an essential element of several therapeutic preparations as it was commonly used by Egyptians, Romans, Greeks and Hindus for treatment of various ailments in ancient times. In the 17th century, iron was used for the treatment of Chlorosis or green disease (a condition occurring as a result of iron deficiency). It was the year 1932 when it was scientifically proven for the first time that inorganic form of iron was required for the synthesis of hemoglobin (6). Iron is extremely important for life and is known to be the second most frequently occurring metal in the earth’s crust. Almost all living forms on earth require iron with the only exception of organisms such as Lactobacilli. It is believed that iron was the catalytic element responsible for the synthesis of macromolecules from hydrogen and carbon dioxide, as a result of which, the earliest life forms were evolved (7).

Iron plays a very important role in several biological functions such as respiration, synthesis of DNA, generation of energy and cell proliferation. It exists in several molecular systems as an essential trace element and has been recognized as a co factor for several cell systems (8). It has been proven that deficiency of iron disturbs not only the psychomotor development among young children but can also alter their normal cognitive functions (9).

## 2 Sources

Although iron is present in both animal and plant foods, yet all foods are not good sources of iron. Foods which do not give good amounts of iron include dairy products and most of the non-green leafy vegetables (10).

### 2.1 Animal sources

Among animal foods, chicken liver contains 12.9 mg of iron per 100 grams while beef gives 3.5 mg of iron for every 100 grams consumed and are considered to be among the richest sources of dietary iron. Lamb provides 2.7 mg while salmon 1.28 mg of iron for every 100 grams serving. The heme content of chicken, beef, and lamb is 81, 77, and 88%, respectively (11). Table 1 provides heme and non-heme iron content in different types of meat (12).

**Table 1:** Heme and non-heme iron content of different types of raw meat (mg/100 g).

Animal species	Total iron (mg/100 g)	Heme iron (mg/100 g)	Non-heme iron (mg/100 g)	Heme iron (%)	Non-heme iron (%)
Beef	1.99 ± 0. 25	1.57 ± 0.29	0.45 ± 0.16	79 ± 9	23 ± 7
Lamb	1.77 ± 0.60	1.35 ± 0.20	0.46 ± 0.19	76 ± 10	27 ± 10
Chicken	0.44 ± 0.28	0.26 ± 0.17	0.19 ± 0.17	66 ± 20	35 ± 21
Turkey	0.92 ± 0.33	0.50 ± 0.33	0.51 ± 0.16	50 ± 18	58 ± 10
Fish	2.07 ± 2.57	0.95 ± 0.89	1.12 ± 1.79	54 ± 20	49 ± 17
Prawn/Shrimp	3.52 ± 4.14	1.91 ± 2.62	2.52 ± 2.87	40 ± 20	75 ± 5

### 2.2 Plant sources

Leafy vegetables are next to follow after spices and herbs. Within the category of spices and herbs, cumin seeds lead by providing 66.36 milligrams of iron per 100 grams. As far as raw legumes are concerned, soybean is at the top of the list, providing 15.70 milligrams per 100 grams (13).

## 3 Iron forms, body stores, and daily requirements

On the basis of classification of dietary sources of iron, two different types of iron are found in human diet, namely non-heme iron and heme iron. Comparably heme proteins are greater sources of iron as compared to non-heme sources (14).

### 3.1 Heme iron

Heme iron is that form of iron which mainly comes from meat and meat products. Major sources of heme iron are myoglobin and hemoglobin which mainly come from chicken, fish, meat, eggs and organ meats such as liver and heart and it is very well absorbed by the human body with a bioavailability of around 10 to 25% (15). The high bioavailability of ferrous (Fe^2+^) form of iron is due to its ability to cross the apical brush border of enterocytes with the help of DMT1 (Divalent Metal Transporter 1) (16).

### 3.2 Non-heme iron

Non-heme iron is predominantly present in dried beans, cereals, pulses, legumes, wheat germ, oysters, egg yolks, and a few fruits and vegetables and it is naturally less absorbed by the human body (17), as its bioavailability is only around 2 to 15% (18). The ferric (Fe^3+^) form is less absorbed by the body because it must be converted into the ferrous form with the help of iron reductase enzyme before it can get absorbed (19).

### 3.3 Iron content in human body

Human bodies contain approximately 3 to 4 grams of iron, 75% of which is bound with heme proteins (myoglobin and hemoglobin) (20). 20% iron is bound with Hemosiderin and Ferritin (storage proteins). 3% of total iron in the body is bound in enzyme systems (catalases, peroxidases and cytochromes) (21).

### 3.4 Daily iron requirements

Iron requirements of infants up to 6 months of age are easily met by the small amounts of iron contained by the human milk. After that, approximately 0.7 to 0.9 milligram of iron per day is needed till the age of 12 months (22). The Recommended Dietary Allowance (RDA) for iron for both genders during early adolescence is 8 mg/day. Growth spurts may increase this requirement by 1–3 mg/day. When they reach 14 to 18 years, the requirement increases to 11 mg/day for males and 15 mg/day for females (23). The RDA for adult males is 8 mg per day and for females 18 mg per day, up to 50 years of age. In the elderly aged 51 years and above the requirement is 8 mg/day for both genders (24).

During pregnancy, the requirements for absorbed iron generally increase from 0.8 milligrams per day in the first trimester to as high as about 7.5 milligrams per day in the third trimester. It has been estimated that the average absorbed iron requirements during the whole pregnancy phase is approximately 4.4 milligrams per day. The absorbed iron during pregnancy is mainly used to fulfill iron requirements of fetus, expand the mass of mother’s erythrocytes, and to compensate for blood losses during delivery (25). The reserves of iron within the infant depend predominantly on the birth weight. In low birth weight infants (2000–2,500 g), the iron requirements have been estimated around 1–2 mg/kg/day. In case of very low birth weight infants (<1,500 g), the infant may need up to 2–3 mg/kg/day of iron. On the other hand, if the birth weight is normal (>2,500 g) no additional iron is required for the first 6 months of life (26).

## 4 Iron cycle

### 4.1 Digestion and absorption of iron

**Figure 1:**
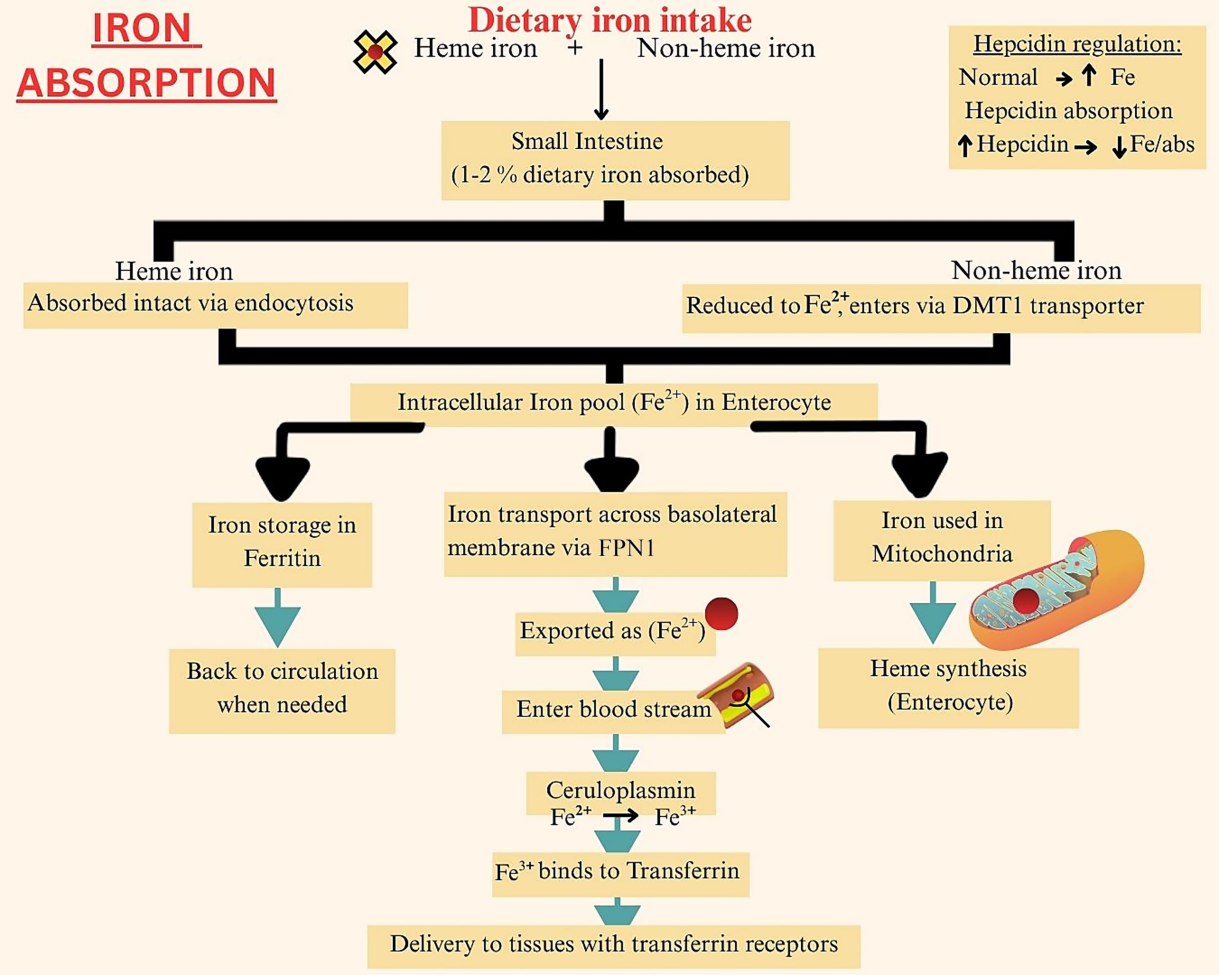
Iron absorption.

The duodenal and jujenal enterocytes of the small intestine are the primary iron absorption site. Of the total dietary iron consumed, only 1–2% gets absorbed per day. Heme iron has a better absorption rate, due to less interaction with other nutrients and dietary factors. Additionally, the intact porphyrin ring of heme iron allows efficient endocytosis of the molecule at the enterocyte brush border (27). Once absorbed by the enterocytes, both heme and non-heme iron enter a common intracellular iron pool. An individual’s iron status determines if the iron will now be stored as ferritin, or if it will be transported to tissues for use via the ferroportin (FPMN1) transporters. These transporters, found in the basal membranes of cells, carry the ferrous form of iron to transferrin protein. This protein converts iron into its oxidized form, which is then taken up by cells with transferrin receptors (28). A portion of the absorbed iron is also transferred to the mitochondria to produce heme molecules. Further iron absorption is aided via the ceruloplasmin molecules in the plasma, which bind extracellular iron to transferrin. Hepcidin released by the liver, also promotes iron absorption and circulation, however at high levels it can lead to iron deficiency anemia as increased hepcidin concentration is associated with iron sequestration (29). Figure 1 provides a step-wise summary of iron absorption.

The figure illustrates the absorption of heme and non-heme iron in the duodenum via HCP1 and DMT1, respectively, and its systemic transport bound to transferrin, with regulation by hepcidin through ferroportin-mediated iron export.

#### 4.1.1 Enhancers of iron absorption

The hydrochloric acid present in gastric secretions converts ferric iron onto ferrous iron, which has a higher absorption rate. In achlorhydria iron absorption was markedly reduced but administering hydrochloric acid increased non-heme iron absorption by 4 times. However, heme iron absorption remained unaffected by hydrochloric acid (30). Ascorbic acid is also evidenced to increase non-heme iron absorption by forming a chelate with iron in the small intestine. The ferric form of iron interacts with ascorbic acid in the acidic pH of the small intestine and the chelate formed remains stable in the duodenum’s alkaline pH (31). Ascorbic acid reduces ferric iron into ferrous iron which has higher bioavailability and then binds with Divalent Metal Transporter 1 (DMT1). The ferric iron forms a complex with ascorbic acid (Fe^3+^− ascorbate complex) which then enters the Caco-2 cells (32).

Meat factors found in animal source foods have a positive impact on various micronutrients including iron status in humans. Meat factors are substances found in animal tissues that enhance iron and other micronutrient absorption from plant foods. For example, the addition of veal or fish meat to a corn based meal significantly increased non-heme iron absorption (33). Research found a 180 and 100% increase in iron absorption from freeze dried beef and chicken consumption. However, consumption of egg albumin was not associated with any increased iron uptake, indicating that protein was the main factor responsible for enhancing iron absorption. This explains why plant based alternatives of meat products despite having high iron content have poor bioavailability of the nutrient (34).

#### 4.1.2 Inhibitors of iron absorption

Phytic acid, also known as Myo-Inositol hexakiphosphate (IP_6_), is known to reduce element bioavailability of iron, zinc and calcium. It binds with these divalent cations forming insoluble complexes, which remain stable and unabsorbed in the small intestine. An example is legumes, which contain high levels of both phytates and these micronutrients, but have poor bioavailability of these elements. Iron absorption increases by 4 to 5 times when phytate concentration is reduced to <0.01 mg/g of isolate (35).

Polyphenols bind with iron to exert their antioxidant activities, as iron is involved in free radical generation. Polyphenols bind with the catechol and galloyl groups present in iron, there bv reducing iron levels required to free radical production. Flavonoids, ellagic acid, curcumin, and iso-flavones, all develop redox-active iron complexes which results in iron depletion (36).

Calcium has the ability to hinder both heme and non-heme iron absorption. The inhibitory action occurs both during iron uptake by the enterocytes, and its transport across the basolateral membrane from enterocyte to plasma. Calcium interferes with the DMT1 receptors and ferroportin protein, both of which are important in iron transportation to small intestine. Calcium supplements hinder non-heme iron absorption, but if given with ascorbic acid food sources such as orange juice, the inhibitory effect of calcium declines significantly (37).

Oxalates act as anti-nutrient compounds for several elements including iron. They have the ability to bind with minerals and form water soluble salts, there by rendering these minerals unavailable for absorption (38).

Animal proteins such as casein have the ability to form casein phosphopeptides (CCPs) which possess chelating properties. The mainly bind with calcium but also play a significant role in inhibiting iron absorption by binding with and forming iron complexes (39). The animal sources derived chelating peptides when bind with iron form stable complexes that are able to withstand gastrointestinal digestion. Even though stomach enzymes such as pepsin and trypsin act on these complexes and break them down while releasing free amino acids, 75% of the complex remains stable and resist luminal iron digestion (40). A summary of different iron enhancers and inhibitors and their mechanism of action is given in Table 2.

**Table 2:** Enhancers and inhibitors of iron absorption.

	Substances/elements	Mechanism of action
Enhancers	Hydrochloric acid (HCl)	• Converts Fe^3+^ to ferrous Fe^2+^ • Improves non-heme iron absorption.
	Ascorbic acid (Vitamin C)	• Reduces Fe^3+^ to Fe^2+^ • Forms stable chelates • Enhances absorption even in alkaline duodenum • Counteracts phytate inhibition
	Meat factors	• Enhance non-heme iron absorption likely due to specific proteins.
	Divalent Metal Transporter 1 (DMT1)	• Facilitates uptake of Fe^2+^ bound to ascorbate complex into enterocytes.
	Dietary phytase (enzyme)	• Breaks down phytic acid, improving iron bioavailability.
Inhibitors	Phytates (IP6)	• Bind Fe^2+^ and form insoluble complexes • Reduce bioavailability
	Polyphenols	• Bind iron (especially Fe^3+^) via catechol/galloyl groups • Form redox-active complexes • Reduce iron absorption
	Calcium	• Inhibits both heme and non-heme iron • Interferes with DMT1 and ferroportin • Hinders transport into plasma
	Oxalic acid/Sodium oxalates (SO)	• Bind with Fe and form large complexes, especially with heme iron • Reduce brush border transport.
	Casein (milk protein)	• Forms casein phosphopeptides (CPPs) that chelate iron and reduce its availability.
	Whey protein	• Acts similarly to casein • reduces iron absorption.
	Fish skin/bones protein hydrolysates	• Contain chelating peptides that bind iron strongly and resist digestion, reducing bioavailability.
	Proton pump inhibitors (PPIs)	• Suppress gastric acid; reduce Fe^3+^ to Fe^2+^ conversion • Lower non-heme iron absorption, especially in deficient individuals.

### 4.2 Transport of iron

After its absorption into the enterocytes, ferrous (Fe^2+^) iron enters the circulation via ferroportin protein. Once in the circulation system, the ferrous iron is oxidized back into its ferric (Fe^3+^) state by ceruloplasmin or hephaestin. This ferric iron then binds with transferrin in the plasma which transports it to various sites for utilization. Transferrin is the major iron binding and transportation protein in healthy individuals. However, in compromised health conditions, the iron in the circulatory system may surpass the trasnferrin protein’s iron binding capacity. The non-transferrin bound iron (NTBI) may then enter the liver and produce toxic effects (41). The NTBI is redox-active and has the potential to trigger the Fenton reaction. This reaction involves ferrous iron (Fe^2+^) and hydrogen peroxide reaction resulting in the production of hydroxyl radicals. These free radicals increase oxidative stress resulting in cell injury and inflammation (42).

The transferrin protein binds two ferric iron with itself and reaches the plasma membrane of cells. Here the transferrin receptor (TfR1) is present which binds with the transferrin protein forming a transferrin- TfR1 complex. This complex is taken up by the cells and reaches the endosomes. These endosomes are acidified to maintain a pH of 5.5–6.0, and the acidic environment degrades the bond between ferric iron and transferrin, thereby releasing iron into the endosome. The enzyme six-transmembrane epithelial antigen of the prostate 3 (Steap3) found on the endosomal membrane reduces the ferric iron to ferrous iron, which then passes the cytoplasmic DMT1 and enters the plasma again to be bound to transferrin again and the cycle continues (43). Cellular iron transport is also influenced by iron regulatory proteins (IRPs) which are a kind of RNA binding proteins. They play a role in regulating mRNA responsible for encoding transferrin, ferroportin, and DMT1. These mRNAs have increased translation rates when IRPs bind with iron-responsive elements (IREs) at the 3′ untranslated region (UTR). Conversely, when IRPs bind at the 5′ UTR, translation into these iron binding proteins is inhibited. If IRPs are not available for mRNA regulation, roquin, another RNA binding protein may perform this function, but this results in destabilization of transferrin receptor mRNA (44). When plasma iron concentrations are higher than usual the IRPs unbind from the IREs as a regulatory mechanism to prevent high cellular iron uptake. This prevents cellular iron toxicity and increases cytoplasmic ferritin stores. Furthermore, the same mRNA responsible for encoding DMT1 and ferroportin receptors also regulate iron metabolism proteins which helps maintain iron status according to cellular demand (45).

### 4.3 Storage of iron

**Figure 2:**
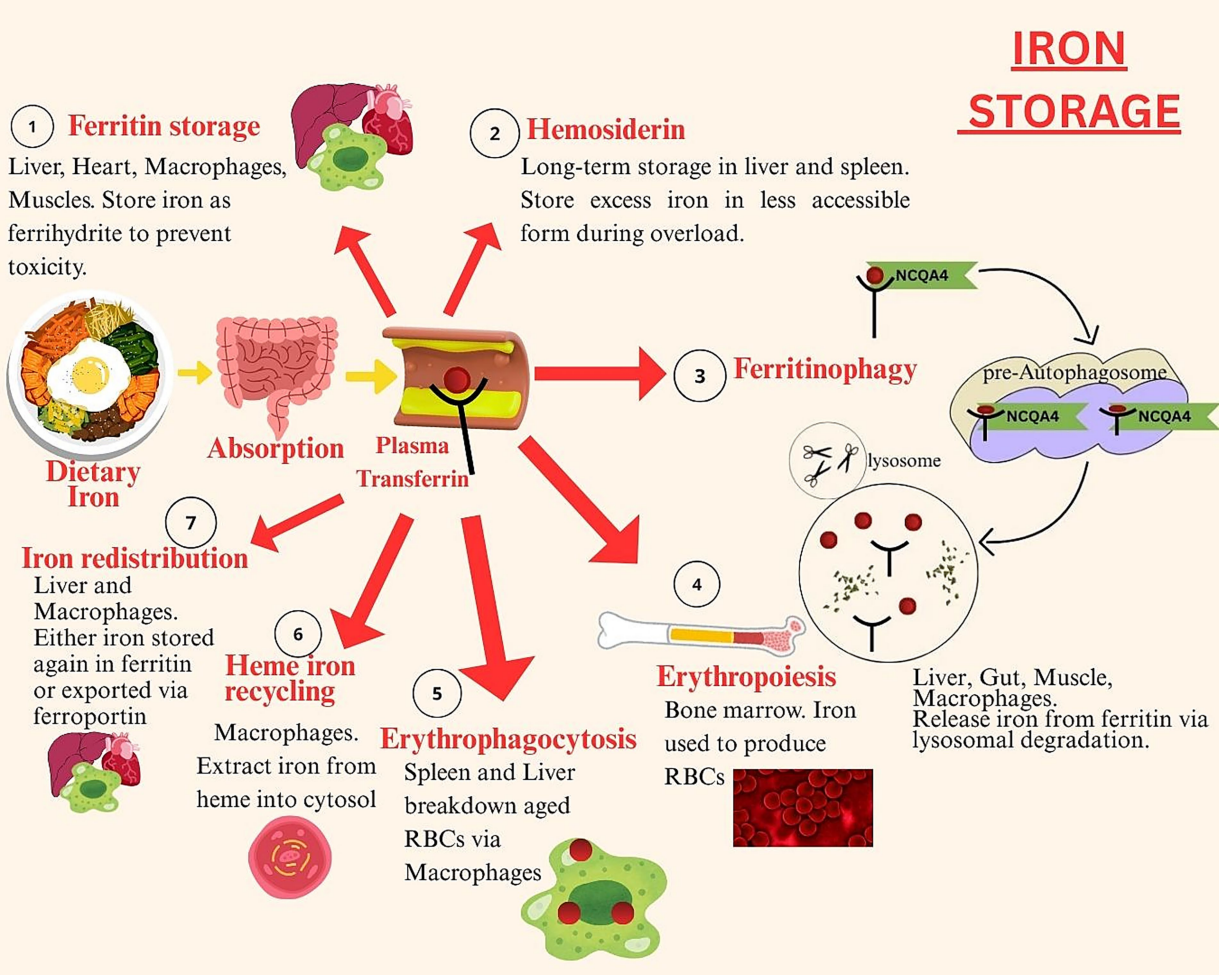
Iron storage.

Iron is stored bound to ferritin protein – the main intracellular iron storage protein in the body. Ferritin’s spherical cage like structure is composed of 24 subunits which can bind up to 4,300 iron atoms per cage. The soluble ferrous iron present in the cytoplasmic iron pool get bound with ferritin, which converts it into its insoluble ferric form. This process involves entry of ferrous iron into the transferrin cage, where oxidation reactions convert it into ferric iron. The ferric iron gets trapped within the transferrin cage and requires reduction to be released. In the last step of iron sequestration by ferritin protein, the ferric iron undergoes denoted mineralization. This involves conversion of ferric iron into mineral ferrihydrite, during which the iron’s core itself acts as catalyzer to the process. Ferritin proteins have 2 subunits; heavy (H) found in heart and light (L) found in the liver. The ferroxidase sites present on the H subunit are involved in ferrous iron oxidation by binding two ferrous irons with itself. These get released into the ferritin cavity, in the form of a diferric peroxo complex ferric intermediate (DFP) (46). Figure 2 shows simplified steps of iron storage in the human body.

The figure depicts the stages of iron storage, including intracellular sequestration as ferritin, long-term storage and mobilization of stored iron based on systemic demand and hepcidin regulation.

Iron stored in ferritin proteins can be released according to the body’s demand via ferritinophagy. This process involves degradation of ferritin in cellular lysosomes which results in release of iron. Within the lysosomes, the ferritin protein binds with nuclear receptor co-activator 4 (NCOA4), a specific cargo receptor and an E3 ubiquitin ligase, HERC2. Upon binding, ferritin breaks down and iron gets released. This iron is then mobilized for erythropoiesis particularly during iron deficiency states. Ferritinophagy is a crucial process in erythrocyte generation as well as in facilitation of dietary iron absorption (47). Around 80% of the plasma iron is stored in bone marrow erythroblasts as erythropoiesis requires 30 mg to 40 mg of iron daily (48). Once erythrocytes are formed and they complete their lifecycle, the iron gets released back into the circulatory system. It gets reused for erythropoiesis in the bone marrow. The erythrocytes are broken down via erythrophagocytosis carried out by reticuloendothelial macrophages, which results in release of iron from heme. This iron is transported through natural resistance associated macrophage protein 1 (NRAMP1) into the cytosol. Within the cytosol it is either stored in ferritin or released back into the circulatory system via ferroportin (49).

## 5 Regulation of systemic iron homeostasis

Systemic iron is regulated via various mechanisms, the most important of which include the hepcidin protein, hypoxia, and sex hormones. Table 3 provides a comprehensive summary of these regulation mechanisms. The hepcidin protein is released by hepatocytes when iron stores are sufficient and further iron absorption can result in iron toxicity. It works by two mechanisms. The first one is known as the endocytosis and degradation mechanism. Hepcidin, a 25 amino acid hormone peptide, hinders iron absorption in the small intestine by binding with ferroportin transporter 1 (FPN1) and causes it to degrade. Hence, blocking the iron absorption process and preventing their release through erythrophagocytosis. Conversely, when iron requirements are high, hepcidin production is reduced via erythroferrone (ERFE). This protein hormone is released during hematopoiesis, by erythroblasts. The hematopoiesis process itself is stimulated by erythropoietin (EPO) (50). The second mechanism involves occlusion of ferroportin molecules by hepcidin, thereby reducing their ability to bind with iron. When hepcidin binds with ferroportin, it causes morphological changes within the molecule, rendering it unable to form linkages with iron as it normally would. However, this process is reversible and once hepcidin concentrations drop during insufficient systemic iron levels, hepcidin unbinds from ferroportin allowing it to return to its original structure. Ferroportin then continues it roles in iron transport as required. Evidence has suggested that hepcidin’s iron lowering effect can last from 24 to 48 h (20).

**Table 3:** Mechanisms of systemic iron regulation.

Systemic iron regulatory mechanism	Key factors	Mechanism of action
Hepcidin protein	Hepcidin, Ferroportin (FPN1), Erythroferrone (ERFE), Erythropoietin (EPO)	1. Binds to FPN1 causing its degradation, blocking iron release. 2. Occludes FPN1, altering its structure to prevent iron transport. 3. Erythropoiesis reduces hepcidin via ERFE to enhance iron absorption.
Hypoxia	Hypoxia Inducible Factors (HIF1α, HIF2α, HIF3α), ARNT, PHD enzymes, EPO, ERFE, BMP5/6/7, SMAD pathway	1. Hypoxia inhibits PHD enzymes, stabilizing HIF1α which activates hypoxia response genes. 2. Stimulates EPO production which triggers ERFE, reducing hepcidin via BMP/SMAD. 3. Low hepcidin boosts intestinal iron absorption for erythropoiesis.
Sex hormones	Testosterone, Progesterone, EGFR, SMAD1/5/	1. Testosterone inhibits SMAD1/5/8 via EGFR, lowering hepcidin and increasing absorption. 2. Progesterone raises hepcidin independently of BMP/SMAD, reducing absorption.

Systemic iron is also regulated by hypoxia inducible factors (HIFs) that associate erythropoiesis with iron homeostasis. Hypoxia stimulates kidneys to produce erythropoietin hormone (EPO). The EPO hormone triggers erythroferrone (ERFE) from erythroblasts. The ERFE promotes erythropoiesis by reducing liver hepcidin levels. This occurs through the Bone Morphogenetic Proteins (BMP), mainly BMP5, BMP6, and BMP7. These proteins activate the BMP/ SMAD (Sma- and Mad-related proteins) pathway involved in hepcidin regulation. Low hepcidin levels promote intestinal iron absorption, required to increase erythrocyte production in bone marrow. Apart from regulating hepcidin, EPO also directly increases iron absorption by binding with its receptor (EPO-R) on enterocytes (51).

Sex hormones, particularly testosterone, and progesterone have also been investigated for their role in systemic iron regulation. The male sex hormone, testosterone, activates the Epidermal Growth Factor Receptor (EGFR) which inhibits the SMAD1/5/8 pathway, resulting in suppressed serum hepcidin levels. This increases iron absorption and explains higher hemoglobin levels in males. The female sex hormone, progesterone, acts inversely and increases hepcidin levels which result in decreased iron absorption. This hormone works independently of the BMP/SMAD pathway, and provides explanation for lower hemoglobin levels in females (52).

## 6 Iron functions and mechanism of action

### 6.1 Role in kinetic reactions

Iron is one of the most essential mineral present in the human body. It is required for several biochemical reactions without which an individual would fail to exist. Both the ferric and ferrous forms of iron are stable within the body and take part in a number of kinetic reactions. Both forms also continuously inter-covert into each other allowing electron transfer and acid–base reactions. Iron’s redox potential size, and ability to bind with ligands help it play vital roles in various biological processes such as oxygen transport, enzymatic reactions, and electron transfer (53).

### 6.2 Hemoglobin formation and oxygen transport

The main function of iron is to form hemoglobin present in erythrocytes and carry oxygen to all parts of the body. Every 1 milliliter of blood contains 0.5 milligram of iron, and loss of blood results in iron deficiency. This is termed as microcytic hypochromic anemia, a condition where erythrocytes becomes smaller in size due to their lower hemoglobin concentration. It results in pallor, breathing difficulties, hypothermia, and various pregnancy, childbirth and post-partum complications. It is also linked with increased risk of maternal deaths. Low iron levels in infancy and childhood increase risk of cognitive dysfunction and poor growth and development (54).

### 6.3 Cellular energy production

Iron plays a crucial role in cellular energy production as it is a key component of heme enzymes, such as cytochromes. These enzymes are required for ATP synthesis as they act as electron carriers in the electron transport chain. Electron transfer in the chain is facilitated by reduction of ferrous to ferric iron. Iron also generates energy through the citric acid cycle, as a part of various iron–sulfur proteins, including succinate dehydrogenase (55).

### 6.4 Cancer fighting and antimicrobial potential

Mixed iron (III) complexes exhibit both anticancer and antibacterial properties. They contain amino acids and isonitrosoacetophenone, both of which are potent anticancer compounds and can be used in development of anticancer drugs. The same compounds also depict antimicrobial activity against both gram positive and gram negative bacteria. The effect is stronger in gram positive bacteria. They also act against some fungi species especially *Candida albicans* (56).

### 6.5 Immune modulation

Iron also plays an essential role in immune modulation. Its deficiency has been linked with impaired B-cell proliferation, poor T-lymphocyte function and disrupted antibody responses. Iron supplements is evidenced to improve immune response in immune-compromised children living in low socio-economic regions. Iron has been evidenced to play a dual role in host defense and pathogen proliferation. In diseases states, the body reduces iron available to pathogens, reducing the spread of diseases. This is regulated through inflammatory cytokines which increase hepcidin production leading to increased iron sequestration. Reducing iron during illness can help promote the body’s natural defense mechanisms (57).

### 6.6 Muscle health maintenance

Iron also plays a vital role in maintaining muscle health and has been linked with sarcopenia prevention. Iron has the ability to bind free radicals thereby reducing oxidative stress. This helps reduce muscle damage and lower risk of sarcopenia, particularly in old age (58). Additionally, iron contributes to energy generation in skeletal muscles via oxygen reducing systems. Iron containing enzymes are required by the skeletal muscle mitochondria to generate energy. Furthermore, oxidative metabolism in these muscles is facilitated by hemoproteins in complexes III and IV, and iron–sulfur clusters (ISC) proteins in complexes I, II and III (59).

### 6.7 Lung homeostasis and protection

Iron helps in maintaining intracellular and extracellular iron balance within the lung, and protects it against oxidative damage. Non-transferrin bound iron (NTBI) ensures sufficient iron transport to lung tissues. It is stored as ferritin within the lung epithelial cells and sequestered by macrophages to prevent free radical formation. During iron overload, the excess iron is stored as ferritin near the airway surface, preventing it from reaching lung tissues and causing oxidative stress (60). Furthermore, iron helps prevent respiratory infections as iron supplementation has been evidenced to reduce both pro-inflammatory cytokines (IL-1β, IL-9, IL-17) and airway hyper-responsiveness. It has been linked with fewer respiratory tract infections and reduced airway dysfunction. It promotes lung protection through innate and adaptive immunity. Iron regulates myeloid cell function, and regulates antimicrobial effectors production, in innate immunity. While in adaptive immunity, iron supports lymphocyte expansion. Both mechanisms boost respiratory infection defense mechanism (61).

### 6.8 Regulation of DNA metabolic pathways, replication and repair

The DNA metabolic reactions require iron as a redox cofactor and for activation of several DNA repair enzymes. The DNA replication process utilizes iron–sulfur clusters to stabilize and activate the replication complexes. During DNA repair, iron dependent redox activity promotes long range charge transfer which helps mismatch detection and correction. This phenomenon helps understand and DNA disorders and conditions like cancer and aging. Iron also promotes genome stability as a crucial component of ribonucleotide reductase enzyme (62).

### 6.9 Role in prenatal development

Iron plays a critical role in supporting erythropoiesis and fetal growth. It is responsible for cell proliferation and differentiation within the embryo. A continuous supply of iron is required to build fetus liver iron stores. Iron regulates placental transport between the visceral endoderm (VE), early placenta, and specialized placenta to prevent reverse iron transfer via placenta back in the mother’s circulation. Iron metabolic genes have a role in normal fetal development as their disruption can cause developmental issues and fetal mortality (63). Iron deficiency also has an impact on neonatal growth. Infants born to iron deficient mothers were 13% lighter in weight as compared to those born to iron replete mothers (64).

### 6.10 Role in preventing cardiovascular disease morbidity and mortality

Iron deficiency is a risk factor for various CVDs, especially heart failure and iron deficiency anemia (IDA) is an independent risk factor for heart failure associated mortality. Correcting the deficiency improves cardiovascular symptoms in patients. Similarly, in pulmonary arterial hypertension (PAH), the risk of iron deficiency is greatly increased and intravenous iron administration can improve exercise capacity in these patients. Cardiac surgery outcomes improve when patients are given iron supplements or blood transfusions. However, iron administration is limited in heart transplant surgery due to its role in T-cell activation which may lead to organ rejection (65).

## 7 Iron overload/toxicity

Iron overload, known as hemochromatosis, is a condition where iron builds up in the blood stream. It occurs either due to genetic causes (hereditary hemochromatosis), iron loading anemia, myelodysplastic syndromes, or repeated blood transfusions. Iron overload is associated with increased oxidative stress which further contributes to health problems (66). Serum iron levels above 350 micrograms/dL or 20–60 mg/kg, 8 to 10 h after iron ingestion indicate iron toxicity in the individual (67). The clinical course of iron toxicity is classified into 5 stages. The first stage lasts from 30 min to 6 h during which gastrointestinal (GI) symptoms such as nausea, abdominal cramping, vomiting, hematemesis, and hematochezia are exhibited. The second stage lasts from 6 h to a day, and the patient shows signs of apparent recovery, this is because the GI tract absorbs the excess iron during this time period. The third stage lasts from 6 h to 3 days and is marked by recurrence of GI symptoms, along with symptoms of shock and metabolic acidosis. Additionally, this stage also depicts hepatic dysfunction, cardiomyopathy, and renal failure. The fourth stage occurs during the 12 to 96 h window and is marked by an increase in aminotransferase levels; an indicator of liver damage. Finally, the fifth stage lasts from 2 to 8 weeks and during this time the GI mucosa starts healing and scarring and obstructions starts to clear (68).

Iron toxicity can affect several organs including the heart, liver, pancreas, and spleen. If iron overload worsens, it can lead to organ damage and complications such as heart and liver failure, fibrosis, metabolic disorders like diabetes, impotence and growth impairment. The severity of organ damage and complications depends on the level of iron accumulation. Lack of proper treatment can lead to irreversible organ damage and consequent complications. Iron toxicity results in cellular damage as evidenced by electron microscopy studies. Iron loaded lysosomes rupture inside the cells which results in mitochondrial swelling. This leads to cytoplasmic vacuolation, nuclear changes, and gradual loss of intra-cellular structures (69). Due to its damaging nature, labile plasma iron (LPI) needs to be suppressed and eliminated from the body. Iron chelating agents are most commonly used in medicine to get rid of excess systemic iron. These agents bind free iron with themselves and prevent it from accumulating in vital organs. The bound iron then leaves the body either via urine or feces. These chelators are used either individually or in combination. These chelating agents aim to balance iron absorption with iron excretion to prevent iron overload (70).

## 8 Losses/excretion of iron

A normal healthy adult experiences 1–2 mg/day of iron losses. Apart from the renal and hepatic iron excretion, it is also lost through intestinal mucosa, and menstruation in females. To maintain iron homeostasis, the iron lost or excreted should be made up for in the same amount. This iron can come from dietary or supplementary sources. Increased iron losses may occur if any of the aforementioned process is dysregulated. Examples include intestinal inflammation, as seen in irritable bowel disease (IBD), leading to increased iron loss from the GI tract. Irregular and heavy menstrual bleeding also results in excessive iron losses (71). Sweating also plays a role in iron excretion, though it is mainly a thermoregulation mechanism. Approximately 1-2 mg of iron can be lost through sweat each day. Iron losses are higher at the beginning of sweating and then at later stages. In case of excessive sweating, as experienced by athletes, iron deficiency may occur. This is further aggravated by hot and humid conditions, which increase sweating and subsequent iron losses. It was reported that a 2 h physical activity sessions over a period of 2 weeks, resulted in 65% decreased iron levels in both genders. Ferritin level dropped by 50% in both males and females after engaging in 4 weeks of exercise (72).

Menstrual iron losses, particularly due to heavy menstrual bleeding are a common issue that effects several women of reproductive age. It is more prevalent in low income countries, and it was reported that 50.6% of Pakistani adolescent girls with heavy menstrual bleeding also had iron deficiency anemia (73). Lactating mothers are also at risk of excessive iron losses, as their iron stores are depleted to meet the infant’s iron requirements after the first 6 months of life. To maintain the quality of breast milk, the mother’s body transfers excessive iron to the breast milk. If the mother was already iron deficient, this can result in depleted iron stores in the mother and inadequate iron for the breastfed infant (74).

## 9 Interaction with other nutrients

### 9.1 Zinc

Both zinc and iron metabolism is deregulated in case of low dietary intake or increased dietary inhibitors such as phytates. Non-heme iron supplementation hinders zinc absorption, but heme iron has no effect on it. Additionally, liquid and ferrous iron have a higher inhibitory impact on zinc absorption. However, this effect can be reduced by taking iron with food, or hisitidine. Iron fortified foods do not negatively impact zinc absorption, and therapeutic iron doses administered to pregnant females also did not influence serum zinc levels. Zinc supplementation, on the other hand leads to minutely reduced ferritin levels which indicates its influence on iron storage. High zinc doses inhibit iron absorption, especially when taken in liquid form. This is because both nutrients compete for the same intestinal transport pathway. Zinc also impacts iron metabolism by indirectly effecting iron regulating proteins, such as hepcidin (75). Combined zinc and iron supplementation is more effective in anemia prevention and management. It helps improve ferritin levels and psychomotor functions in infants. However, this effect is dose sensitive as higher zinc doses inhibit iron absorption (76).

### 9.2 Calcium

Calcium has been deemed as an inhibitor of iron absorption. It does so via two mechanisms; firstly calcium may internalize the divalent metal transporter 1 (DMT1). This results in reduced erythrocyte iron uptake. Secondly, calcium may interfere with iron’s transfer across the erythrocyte basolateral membrane. The first mechanism is however compensated by various iron homeostatic process, making is less effective in hindering iron absorption. Evidence is still lacking on what the calcium threshold dose is for inhibiting iron absorption. Furthermore, there is mixed evidence on whether calcium has the ability to chronically impact iron status within the body (77). The inverse relationship between calcium and dietary iron absorption holds true for both calcium supplements and dairy products. Dietary calcium reduction can enhance iron absorption from meals, but that would result in inadequate calcium to meet daily needs. This issue is amplified in vulnerable population groups, such as children and elderly, who require a balanced amount of both nutrients. Recommendations suggest spacing out high calcium and high iron foods, which is not always a feasible option. Further research is needed on calcium’s inhibitory effect on iron and its clinical implications (78).

### 9.3 Copper

Both iron and copper are essential trace elements involved in various redox reactions as cofactors. Because of this their homeostasis is tightly controlled at both cellular and systemic levels to prevent excess free radical generation. Iron and copper have interconnected metabolic pathways and impact each other’s absorption and storage. Iron absorption has been discussed in detail in this review. Copper homeostasis is maintained via chaperones in cells and metallothionein (MT) binds any excess copper. Gut reabsorption of copper is regulated through biliary secretions. Copper helps prevent iron deficiency by either enhancing iron absorption or erythropoiesis. This was evidenced as early as the mid nineteenth century, when women working in copper factories were less likely to develop iron deficiency anemia. While copper helps maintain iron levels during periods of deficiency, high iron can disrupt copper levels in the body (79). Evidence has linked high iron intake with copper deficiency diseases, thereby developing a link between high iron intakes and increased copper requirement. This interaction is significant clinical implications during pregnancy as low copper levels can impair fetal growth and development. Therefore, iron supplements and iron fortified foods are recommended to include copper as well, to make up for copper losses that occur due to iron intake (80).

### 9.4 Folate

Folate interacts with iron at multiple sites and influences iron absorption, storage, transport and utilization. Duodenal iron transporters are not directly impacted by folate, rather it affects iron bioavailability in various organs. Furthermore, folate regulates iron transporters such as DMT1, thereby preventing excessive hepatic iron accumulation. This reduces risk of oxidative stress and hepatic damage. Folate deficiency may also lead to iron being trapped in storage rather than being used to synthesize hemoglobin. Both folate and iron are required to improve hemoglobin level and mean corpuscular volume (MCV). This indicates improved erythrocyte formation. Folate also influences hepcidin levels to prevent iron toxicity. Iron and folic acid supplements are less effective at improving pregnancy outcomes and reducing anemia risk, as compared to multiple micronutrient supplementation (81). Iron may also play a role in regulating folate metabolism but the exact mechanism is unknown. Both the level of iron and folate deficiency as well supplementation dose, determines the mutual influence of both nutrients of their respective transport proteins (82).

### 9.5 Vitamin A

Vitamin A is involved in iron metabolism as an influencer of hepcidin expression, and can increase or decrease its levels based on other contributing factors. It elevates hepcidin levels through raising pro-inflammatory cytokines (IL-6, IL-1β), responsible for hepcidin production. BMP6 protein levels are also elevated during vitamin A deficiency, and this leads to enhanced hepcidin transcription rates. Vitamin A also interferes with erythropoiesis and impairs erythrocyte production. This leads to reduced levels of and malformed erythrocytes, as well as heme accumulation in the spleen. Through these mechanisms, vitamin A deficiency disrupts iron absorption and utilization and worsens iron deficiency (83). Vitamin A also influences hematopoiesis, iron mobilization, and gene expression. Additionally, vitamin A precursor beta carotene reduces ferritin levels and prevents iron sequestration within cells by increasing Fpn1 expression. Vitamin A also elevates mRNA levels of iron regulatory protein 2 (IRP2) and ferritin. This traps iron within enterocytes and inhibits its transport to target cells. Evidence suggests that taking both iron and vitamin A supplementation together helps prevent iron deficiency anemia (84).

## 10 Indicators of iron deficiency and overload

Iron homeostasis in the body is tightly regulated, and deficiency of iron is evidenced by a number of indicators. Table 4 provides a summary of these indicators, their usage, and limitations. Iron status is assessed in three main body compartments: iron storage, transport iron, and functional iron. There are iron indicators specific to each compartment. Stored iron status is assessed via stainable bone marrow iron and serum ferritin. Stainable bone marrow is the gold standard for stored iron assessment but due to difficulty in withdrawing it, it is not used widely. Iron stores depletion depicts the first stage of iron deficiency, but with no erythropoietic effects as of yet. Transport iron status is indicated by transferrin saturation, erythrocyte protoporphyrin, and soluble serum transferrin receptors. These indicators depict whether sufficient iron is being transported for erythropoiesis. If this demand is not being met, the second stage of iron deficiency settles in, and hemoglobin level decline remarkably. Lastly, functional iron status indicators include hemoglobin and hematocrit levels. Hemoglobin is the functionally vital indicator of iron deficiency, especially iron deficiency anemia. Hematocrit levels provide no additional information about functional iron beyond hemoglobin. Some important confounders of iron status indicators include inflammation, lead poisoning, smoking, pregnancy, dehydration, high altitude, and increased erythropoietic activity (85). All these factors including some clinical factors such as liver disease, disrupted circadian rhythms, autoimmune diseases, thalassemia, malignancy, cardiomyopathies, and certain nutritional deficiencies can all effect iron related indicators. Furthermore, socio-demographics such as gender, age, socio-economic status, and geography all impact iron indicators. It is important to differentiate iron deficiency occurring from dietary restriction or from erythropoietin use. When iron is restricted via diet, iron is available in the bone marrow but hepcidin blocks its release from macrophages, and therefore, iron is not incorporated into erythroid precursors. This is common in chronic diseases such as infections, cancer, and inflammation. Iron deficiency can also occur in individuals undergoing treatment with erythropoiesis-stimulating agents (ESA), such as in renal failure or cancer induced anemia. In this scenario the ESH stimulate erythrocyte production which increase demand for iron, leading to relative or functional iron deficiency (86).

**Table 4:** Iron assessment indicators.

Indicator of iron assessment	Application	Limitations
Stainable bone marrow iron	Gold standard for stored iron assessment	Invasive and not widely used
Serum ferritin	Assesses stored iron	Affected by inflammation, liver disease, etc.
Transferrin saturation	Indicates transport iron	May be influenced by inflammation or nutritional status
Erythrocyte protoporphyrin	Assesses transport iron availability for erythropoiesis	Less commonly used
Soluble serum transferrin receptors (sTfR)	Reflects iron demand and transport	Useful in presence of inflammation
Hemoglobin	Primary indicator of functional iron and anemia	Affected by non-iron factors like hydration, altitude, etc.
Hematocrit	Assesses functional iron	Does not provide more info than hemoglobin
Reticulocyte hemoglobin content (CHr)	Used in inflammation or for anemia classification	Not commonly used in all settings
Mean corpuscular volume (MCV)	Helps classify anemia type	May be altered by other nutrient deficiencies
Red cell distribution width (RDW)	Used to classify anemia	Nonspecific, but helpful when combined with other tests

Out of the numerous iron status indicators, it is not possible to consider a single one as a gold standard to diagnosing iron deficiency. It is proposed to combine various indicators to get a more accurate reading of an individual’s iron levels and to mitigate the confounding effect of various clinical conditions as discussed above. Guidelines recommend starting iron assessment with the basic iron status indicators and then moving to more specific ones. During the initial assessment, hemoglobin and serum ferritin should be assessed as they are cost-effective and efficient measures to diagnose iron deficiency anemia. In the second step, if inflammation in present, reticulocyte hemoglobin content (CHr) and soluble transferrin receptor (sTfR) should be used as iron status indicators. For classification of anemia sub-type, CHr, mean corpuscular volume (MCW), and red cell distribution width (RDW) can be used. Once biochemical assessment has confirmed and classified iron deficiency, the next step is to start dietary intervention. This may include iron supplementation and/or an iron rich diet. In case of severe deficiency, iron replacement therapy (IRT) is administered either orally or intravenously (87).

## 11 Conclusion

Iron is a significant micronutrient in terms of its association with human health, ranging from its nutrition acquisition to its major role in various metabolic pathways. It is a vital part of multiple bodily functions including oxygen transport, immune modulation, and maintaining cellular homeostasis. It is regulated through various factors including hepcidin, erythropoietic demand, and inflammatory mediators including IL-6, IL-22, interferon, Activin B, and BMP2. If iron’s regulation is disrupted, it can result in various clinical conditions ranging from iron deficiency anemia to iron overload. A deeper understanding of iron’s regulatory pathways and its systemic functions can help pave the way for targeted nutrition and therapeutic strategies in iron-related disorders.
